# Color Temperature Tunable White-Light LED Cluster with Extrahigh Color Rendering Index

**DOI:** 10.1155/2014/897960

**Published:** 2014-01-23

**Authors:** Minhao Zhang, Yu Chen, Guoxing He

**Affiliations:** ^1^Department of Applied Physics, Dong Hua University, 1882 Yan'an Road (W), Shanghai 200051, China; ^2^Shanghai Yaming Lighting Co., Ltd., 1001 Jiaxin Road, Jiading District, Shanghai 201801, China

## Abstract

The correlated color temperature (CCT) tunable white-light LED cluster with extrahigh color rendering property has been found by simulation and fabricated, which consists of three WW LEDs (CCT = 3183 K), one red LED (634.1 nm), one green LED (513.9 nm), and one blue LED (456.2 nm). The experimental results show that this cluster can realize the CCT tunable white-lights with a color rendering index (CRI) above 93, special CRI R9 for strong red above 90, average value of the special CRIs of R9 to R12 for the four saturated colors (red, yellow, green, and blue) above 83, and luminous efficacies above 70 lm/W at CCTs of 2719 K to 6497 K.

## 1. Introduction

Semiconductor white light-emitting diodes (LEDs) have attracted a great deal of attention in solid-state lighting applications. Due to their potential for substantial energy savings, high efficiency, small size, and long lifetime, it has been projected that LEDs will broadly replace conventional incandescent and fluorescent lamps for general lighting in the future. It has been reported that a new class of light-detecting retinal cells, the ganglion cells, send their signals to the brain's circadian clock [1, 2]. Inappropriate lighting conditions were shown in mammals to upset the body chemistry and to lead to deleterious health effects, including cancer [3]. Thus, circadian light sources with tunable color temperature would be beneficial to human health, well-being, and productivity. Furthermore, such circadian lights could lead to a reduced dependence on sleep-inducing pharmaceuticals. For this reason, sources replicating the sun's high color temperature during the midday period and low color temperatures during early morning and at night would be a wonderful illumination source, given that we humans adapted to such a circadian source during evolution. Some correlated color temperature (CCT) tunable white-light LED clusters have been discussed [4–15]. The challenge in the design of white light LED clusters with CCT tunable consists of achieving excellent color rendering index (CRI) values [16] over a reasonable range of color temperatures while at the same time maximizing their luminous efficacies (LEs). One problem with the CRI is that it can give fairly high scores to sources that render some saturated object colors very poorly [17, 18]. In particular, the report from CIE Technical Committee TC 1–62 “Color rendering of white LED light sources” [19] summarizes several problems of the CRI when applied to white LED sources. The CRI score does not correlate well with visual evaluation in many cases. One of reasons was assumed to be the different order of magnitude of the color differences occurring if the reflecting samples are illuminated by a white LED light source and by other light sources, due to the peculiar spectral power distributions of the white LED light sources “interacting” with the spectral reflectance of the test-color samples. This is especially noticeable for the case of test-color sample no. 9 of the CIE method, which is a strong red test-color sample. An improved indicator, color quality scale (CQS), has recently been proposed by National Institute of Standards and Technology [20]. It was found that the CQS provides scores consistent with the CRI for the most recent phosphor type LED products, RGBA LEDs, and traditional discharge lamps [20]. So the CRI as a metric for evaluating the color rendering abilities of white-light sources is suitable for the white LED cluster with the phosphor-conversion LED (PC LED).

In this paper, a CCT tunable white-light LED cluster with extrahigh color rendering (CRI > 93 and R9 > 90) has been found by simulation. The predicted and measured results are presented.

## 2. Model for LED Spetra

A model for LED spectra at different drive currents was developed. The relative SPD of the single color LED, S_LED_(*λ*, *λ*_0_, Δ*λ*), was given by

(1) SLED(λ,λ0,Δλ)=exp⁡⁡[−k1(λ−λ0)2(Δλ)2]×cosh⁡[k2(λ−λ0)2(Δλ)2],

where

(2) Δλ={Δλ1,(λ<λ0)Δλ2,(λ≥λ0),ki={ki1,(λ<λ0)ki2,(λ≥λ0),   (i=1,2),

where  *λ*_0_ refers to peak wavelength, Δ*λ*_1_ refers to the left-half-spectral width which is 2∫_380 nm_^*λ*_0_^S_LED_(*λ*)*dλ*, and Δ*λ*_2_ refers to the right-half-spectral width which is 2∫_*λ*_0__^780 nm^S_LED_(*λ*)*dλ*. *k*_*i*_ (*i* = 1,2) are characteristic parameters of spectral shape. The units of peak wavelength and half-spectral width are nanometers.

The relationships of *λ*_0_ and drive current *I*_*F*_, Δ*λ*_*i*_, and *I*_*F*_ were given by (3), and (4), respectively [10], as follows:

(3) $$\begin{array}{c} \lambda_{0}\left(I_{F}\right)=A_{\lambda_{0}}\exp\left(B_{\lambda_{0}}I_{F}\right)+C_{\lambda_{0}} \end{array}$$

(4) $$\begin{array}{c} \Delta \lambda_{i}\left(I_{F}\right)=A_{\Delta \lambda i}+B_{\Delta \lambda i}I_{F}, \end{array}$$

where *A*_*λ*_0__, *B*_*λ*_0__, and *C*_*λ*_0__are function parameters of *λ*_0_(*I*_*F*_); *A*_Δ*λi*_ and *B*_Δ*λi*_ are function parameters of Δ_*λi*_(*I*_*F*_). The unit of drive current is milliamperes.

The relative SPD of phosphor-coated white LED, *S*_*W*_(*λ*), was given by [10]

(5) $$\begin{array}{c} S_{W}\left(\lambda \right)=S_{B}\left(\lambda \right)+S_{F}\left(\lambda \right), \end{array}$$

where *S*_*W*_(*λ*),  *S*_*B*_(*λ*), and *S*_*F*_(*λ*) are the white spectra, blue spectra, and the fluorescence spectra of the white LED, respectively. *S*_*B*_(*λ*) can be expressed by (1). *S*_*F*_(*λ*) can be determined by ∑_380 nm_^475 nm^[*S*_*W*_(*λ*) − *S*_*B*_(*λ*)]^2^→ min. Equations (3) and (4) can be applied to blue spectra *S*_*B*_(*λ*).

The relationship of the fluorescent spectra and the drive current, *S*_*F*_(*λ*, *I*_*F*_), was given by [10]

(6) $$\begin{array}{c} S_{F}\left(\lambda ,I_{F}\right)=S_{F}\left(\lambda ,I_{F\max}\right)+A_{F}\exp\left(B_{F}I_{F}\right), \end{array}$$

where *A*_*F*_ and *B*_*F*_ are function parameters of *S*_*F*_(*λ*, *I*_*F*_). To predict the drive current and input power *P*_in_ of LED at given luminous flux Φ, the relationships of *I*_*F*_ and Φ, *P*_in_, and Φ were given by (7) and (8), respectively, as follows:

(7) $$\begin{array}{c} I_{F}\left(\Phi \right)=k_{I}\Phi^{\gamma }\left(1+c_{I}\Phi^{2}\right) \end{array}$$

(8) $$\begin{array}{c} P_{\text{in}}\left(\Phi \right)=k_{P}\Phi^{\gamma }\left(1+c_{P}\Phi^{2}\right), \end{array}$$

where *k*_*I*_, *c*_*I*_, and *γ* are function parameters of *I*_*F*_(Φ); *k*_*P*_, *c*_*P*_, and *γ'* are function parameters of *P*_in_(Φ). The units of luminous flux and input power are lumens and watts, respectively.

**Figure 1:**
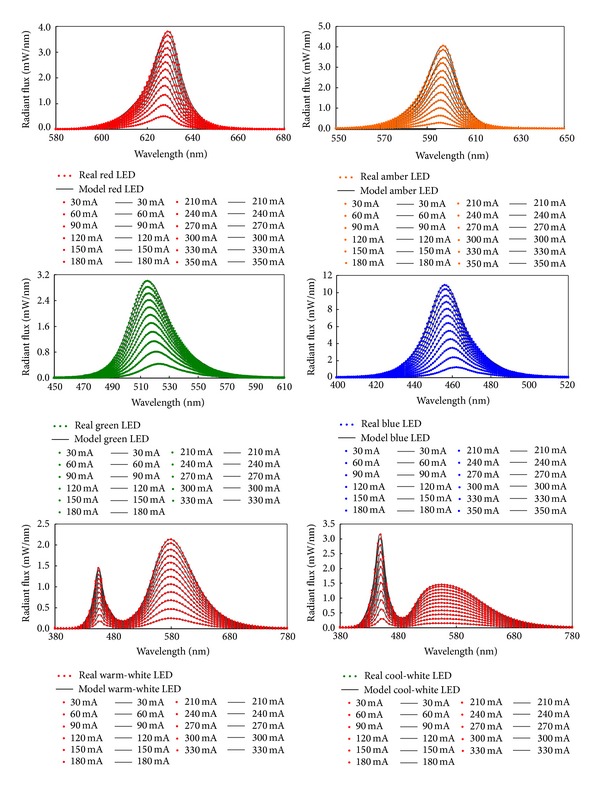
SPDs of model and real LEDs (red, amber, green, blue, warm-white, and cool-white) at different drive currents.

The SPDs of model and real LEDs (red, amber, green, blue, warm-white, and cool-white) at different drive currents are shown in Figure 1. The average Chi-square per degree of freedom (Chi^2^/DoF) for the model and real SPDs of these LEDs at different drive currents is shown in Table 1. The results show that the SPDs of model LEDs are very close to those of real LEDs at different drive currents.

## 3. Simulation and Realization CCT Tunable White-Light Cluster

To analyze the possible performance of the CCT tunable white-light LED cluster, the simulation program has been developed according to the principle of additive color mixture [10]. The simulation program can predict not only the relative SPD, chromaticity coordinates, but also numbers of LED (*N*), drive currents (*I*_*F*_), the input power (*P*_in_), the luminous flux (Φ), and the luminous efficacy (*η*) according to requirements of CRI, R9, CCT, and the distance from the Planckian locus on the CIE 1960 uv chromaticity diagram (dC), with polarity, plus (above the Planckian locus) or minus (below the Planckian locus) [15].

**Figure 2:**
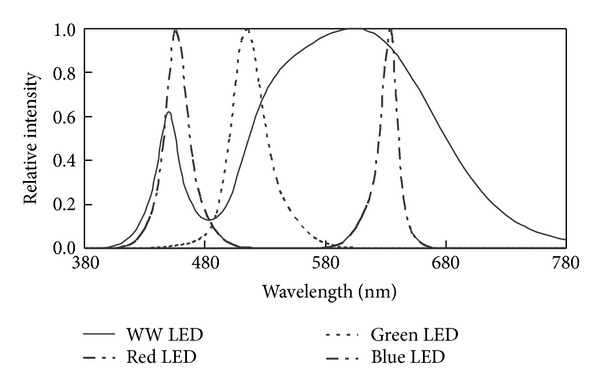
Relative SPDs of WW, red, green, and blue LEDs at drive current of 350 mA.

**Figure 3:**
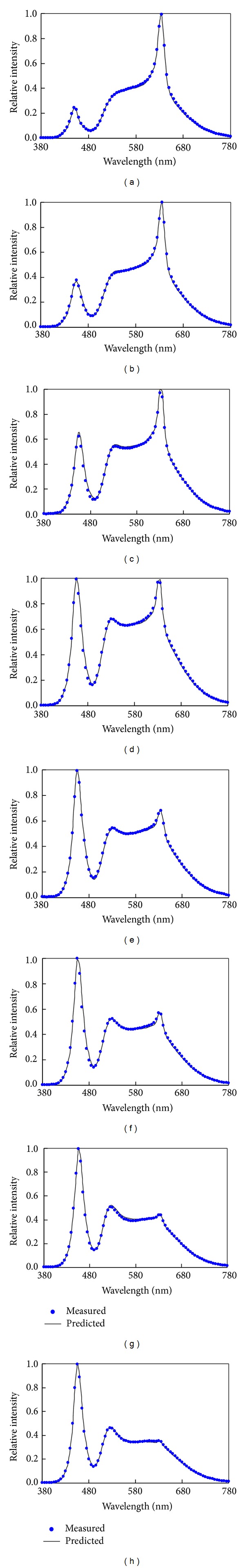
Predicted and measured SPDs of the white light-LED cluster at different CCTs: (a) CCT = 2719 K; (b) CCT = 3028 K; (c) CCT = 3458 K; (d) CCT = 3983 K; (e) CCT = 4537 K; (f) CCT = 5012 K; (g) CCT = 5723 K; (h) CCT = 6497 K.

The CCT tunable white-light LED cluster with extrahigh color rendering has been found by simulation analysis, which consists of three warm-white (WW) LEDs (excited wavelength *λ*_0_ = 450.5 nm, CCT = 3183 K, Φ = 93.2 lm, *P*_in_ = 1.15 W, and *η* = 81.0 lm/W at *I*_*F*_ = 350 mA), one red LED (*λ*_0_ = 634.1 nm, Φ = 51.6 lm, and *P*_in_ = 0.83 W at *I*_*F*_ =350 mA), one green LED (*λ*_0_ = 513.9 nm, Φ = 61.5 lm, and *P*_in_ = 1.24 W at *I*_*F*_ = 350 mA), and one blue LED (*λ*_0_ = 456.2 nm, Φ = 15.3 lm, and *P*_in_ = 1.18 W at *I*_*F*_ = 350 mA). The SPDs, the luminous flux, and the input power of the WW LED, red, green, and blue LEDs at drive currents of 30~350 mA are measured by an automated photometric/radiometric measurement setup and a power meter at an ambient temperature (Ta) of 25°C. The relative SPDs of WW, red, green, and blue LEDs at drive current of 350 mA are shown in Figure 2. An opal bulb is used to mix the light from the single color LEDs to generate uniform white light. The drive circuit is stable and can be precisely controlled, so that it can drive the LED cluster stably and properly according to the optimal drive currents. The predicted and measured SPDs of the white-light LED cluster at different CCTs are shown in Figure 3. The results show that the SPDs of predicted LEDs are very close to those of measured LEDs at different drive currents. The predicted and measured color rendering property and the luminous efficacy of this cluster at an ambient temperature (*T*_*a*_) of 45°C are shown in Table 2. The R(9–12) in Table 2 is the average value of the special color rendering indices R9 to R12 of the four saturated colors (red, yellow, green, and blue). Table 2 indicates that the predicted results are very close to the measured values. The experimental results show that this cluster can realize CCT tunable white-light with a CRI above 93, R9 above 90, R(9–12) above 83, and a luminous efficacy above 70 lm/W at CCTs of 2719 K to 6497 K. Furthermore, their special CRIs of R14 and R15 corresponding to the colors of the skin on the face of European and Chinese women are also very high (R14 > 89 and R15 > 94). R14 and R15 are especially important for interior lighting.

## 4. Conclusion

The white-light LED cluster consisting of the WW (CCT = 3183 K), red (634.1 nm), green (513.9 nm), and blue (456.2 nm) LEDs can realize the CCT tunable white-light with a CRI above 93, R9 above 90, R(9–12) above 83, and a luminous efficacies above 70 lm/W at CCTs of 2719 K to 6497 K.

## Figures and Tables

**Table 1:** Average Chi-square per degree of freedom (Chi^2^/DoF) for the model and real SPDs of these LEDs at different drive currents.

LED	Red	Amber	Green	Blue	Warm-white	Cool-white
Average Chi^2^/DoF × 10^−5^	1.7	1.3	4.5	1.3	1.2	2.0

**Table 2:** The predicted and measured results of the white-light LED cluster at *T*_*a*_ = 45°C.

		Predicted results								Measured results							
	CCT (K)	2703	2952	3431	3922	4490	4976	5715	6547	2719	3028	3458	3983	4537	5012	5723	6497
WW LED	*I*_*F*_ (mA)	273	268	260	250	239	229	214	203	273	268	260	250	239	229	214	203
Red LED	*I*_*F*_ (mA)	156	126	87	60	44	34	20	10	156	126	87	60	44	34	20	10
Green LED	*I*_*F*_ (mA)	27	56	88	115	140	160	198	213	27	56	88	115	140	160	198	213
Blue LED	*I*_*F*_ (mA)	0	12	53	90	129	154	179	210	0	12	53	90	129	154	179	210
	dC × 10^−3^	+1.2	+1.6	−0.9	−2.6	−4.9	−5.2	−3.0	−3.3	−1.1	−2.6	−3.6	−4.5	−4.7	−5.4	−4.6	−4.9
	CRI	94	93	94	95	96	96	95	95	93	93	94	94	95	94	94	94
	R9	94	92	90	93	98	98	94	91	90	94	92	95	96	95	96	93
	R(9–12)	85	82	83	84	86	85	86	85	84	85	83	84	85	85	84	83
	R14	89	89	91	92	93	94	94	94	89	90	90	91	91	90	91	91
	R15	99	97	98	99	97	97	95	94	98	99	99	98	95	95	95	94
	Φ (lm)	261	260	258	254	250	245	239	232	256	250	262	254	245	244	234	228
	*η* (lm/W)	87	87	84	82	79	76	73	71	85	84	85	85	78	76	73	70
